# Dimensional Control in Polyoxometalate Crystals Hybridized with Amphiphilic Polymerizable Ionic Liquids

**DOI:** 10.3390/ma12142283

**Published:** 2019-07-16

**Authors:** Toshiyuki Misawa, Jun Kobayashi, Yoshiki Kiyota, Masayuki Watanabe, Seiji Ono, Yosuke Okamura, Shinichi Koguchi, Masashi Higuchi, Yu Nagase, Takeru Ito

**Affiliations:** 1 Department of Chemistry, School of Science, Tokai University, 4-1-1 Kitakaname, Hiratsuka 259-1292, Japan; 2 Department of Applied Chemistry, School of Engineering, Tokai University, 4-1-1 Kitakaname, Hiratsuka 259-1292, Japan

**Keywords:** inorganic–organic, hybrid crystal, ionic liquid, amphiphilic, polyoxometalate

## Abstract

Ionic liquids are an important component for constructing functional materials, and polyxometalate cluster anion is a promising partner for building inorganic–organic hybrid materials comprising ionic liquids. In such hybrid materials, the precise control of the molecular arrangement in the bulk structures is crucial for the emergence of characteristic functions, which can be realized by introducing an amphiphilic moiety into the ionic liquids. Here, an amphiphilic polymerizable imidazolium ionic liquid with a methacryloyl group was firstly hybridized with polyoxometalate anions of octamolybdate ([Mo_8_O_26_]^4−^, Mo_8_) and silicotungstate ([SiW_12_O_40_]^4−^, SiW_12_) to obtain inorganic–organic hybrid crystals. The polymerizable ionic liquid with a octyl chain (denoted as MAImC_8_) resulted in the formation of anisotropic molecular arrangements in the bulk crystal structure, which was compared with the hybrid crystals composed from the polymerizable ionic liquid without a long alkyl chain (denoted as MAIm). Rather densely packed isotropic molecular arrangements were observed in the hybrid crystals of MAIm–Mo_8_ and MAIm–SiW_12_ due to the lack of the amphiphilic moiety. On the other hand, using the amphiphilic MAImC_8_ cation gave rise to a honeycomb-like structure with the Mo_8_ anion and a layered structure with the SiW_12_ anion, respectively.

## 1. Introduction

Ionic liquid molecules attract extensive attention from researchers owing to their characteristic properties such as conductivity, catalysis, and separation abilities [1,2,3,4,5,6,7,8,9,10,11]. These properties are quite attractive for the construction of functional materials. The hybridization of ionic liquids as organic components with other inorganic counter parts is effective for building up functional inorganic–organic hybrid materials. To select inorganic counter parts for the hybridization is crucial for improving the thermal stability and other properties.

Polyoxometalate (POM) cluster anions are effective inorganic components for constructing inorganic–organic hybrid materials due to their characteristic physicochemical properties [12,13,14,15,16,17,18,19,20]. Hybridization of ionic liquids with POMs has been investigated, and several functional hybrids have been successfully realized such as solid electrolytes or catalysts [21,22,23,24,25,26,27,28]. These ionic liquid–POM hybrids sometimes have POM arrangements in a disordered manner, which may be a drawback for exploiting their characteristics in solid-state hybrid materials. The dimensional control of the components in the solid state is quite significant for the emergence and control of characteristic properties [29]. Introducing amphiphilic moieties, such as a long alkyl chain [30,31,32,33,34,35,36] in ionic liquids is an effective way of constructing single-crystalline ordered structures in the ionic liquid–POM hybrids [37,38,39,40,41].

**Figure 1:**
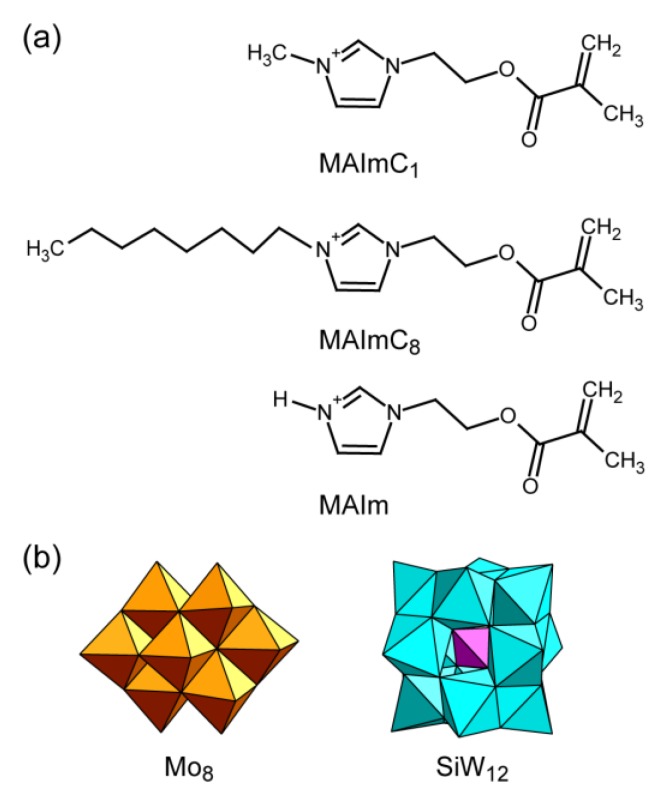
Molecular structures of the components: (**a**) polymerizable ionic liquids of MAImC_1_, MAImC_8_, and MAIm; (**b**) polyoxometalates of β-type octamolybdate (Mo_8_) and dodecatungstosilicate (SiW_12_) anions.

Recently, we designed a polymerizable ionic liquid cation with an imidazolium moiety with a methacryloyl group ([{CH_2_=C(CH_3_)COO(CH_2_)_2_}C_3_H_3_N_2_(CH_3_)]^+^, denoted as MAImC_1_, Figure 1a), and successfully synthesized inorganic–organic hybrid monomers and polymers with several POM anions [42,43,44]. These MAImC_1_–POM hybrids were isolated as single crystals with clarified structures, and behaved as monomers of hybrid polymers exhibiting high conductivity as solid electrolytes [44]. In the MAImC_1_–POM hybrid monomers, the POM arrangements in the bulk crystal structures were isotropic due to the small molecular size of the MAImC_1_ cation. Introducing an amphiphilic moiety into MAImC_1_ will give rise to a more anisotropic molecular arrangement of POM anions derived from the structure-directing ability of the amphiphilic moiety [30,31,32]. 

Here we report the first syntheses of POM hybrid crystals with amphiphilic polymerizable ionic liquids. The amphiphilic polymerizable ionic liquid is a MAImC_1_ derivative with a long octyl chain instead of a methyl group ([{CH_2_=C(CH_3_)COO(CH_2_)_2_}C_3_H_3_N_2_(C_8_H_17_)]^+^, denoted as MAImC_8_, Figure 1a). Another MAImC_1_ derivative without a methyl group ([{CH_2_=C(CH_3_)COO(CH_2_)_2_}C_3_H_4_N_2_]^+^, denoted as MAIm, Figure 1a) was also utilized to hybridize with POM anions for comparison. The POM anions hybridized were β-type octamolybdate ([Mo_8_O_26_]^4–^ (Mo_8_), Figure 1b) and dodecatungstosilicate ([SiW_12_O_40_]^4–^ (SiW_12_), Figure 1b), both of which are anions with a 4− charge. Hybrid crystals with Mo_8_ and SiW_12_ have been reported previously [42,44]. The hybrid crystals with MAImC_8_ contained anisotropic arrangements of POM anions in their bulk crystal structures due to the presence of an amphiphilic moiety, while hybrid crystals with MAIm exhibited isotropic POM arrangements.

## 2. Materials and Methods 

### 2.1. Materials and General Methods

All chemical reagents were purchased from FUJIFILM Wako Pure Chemical Corporation (Osaka, Japan) and Tokyo Chemical Industry Co., Ltd. (TCI, Tokyo, Japan). The starting polymerizable ionic liquid of [{CH_2_=C(CH_3_)COO(CH_2_)_2_}C_3_H_3_N_2_] (denoted as MAIm–N) was synthesized as a neutral compound, and [{CH_2_=C(CH_3_)COO(CH_2_)_2_}C_3_H_3_N_2_(C_8_H_17_)]Br (MAImC_8_·Br) were synthesized as bromide salts according to previous studies (Figure S1) [42,43,44]. 

Infrared (IR) spectra were recorded on a Jasco FT/IR-4200ST spectrometer (JASCO Corporation, Tokyo, Japan) by the KBr pellet method. Powder X-ray diffraction (XRD) patterns were measured with a Rigaku MiniFlex300 diffractometer (Rigaku Corporation, Tokyo, Japan) by using Cu Kα radiation (*λ* = 1.54056 Å) at ambient temperature. CHN (carbon, hydrogen, and nitrogen) elemental analyses were performed with a PerkinElmer 2400II elemental analyzer (PerkinElmer, Waltham, MA, USA). 

### 2.2. Syntheses of Polyoxometalate Hybrids with Polymerizable Ionic Liquids

#### 2.2.1. Synthesis of MAIm–Mo_8_

As-prepared MAIm–Mo_8_ (denoted as **1**) was initially precipitated by a cation exchange reaction. Na_2_MoO_4_·2H_2_O (1.0 g, 4.1 mmol) was dissolved in 10 mL of H_2_O, and then the pH was adjusted to 3.8 by 6 M HCl. To the obtained homogeneous solution was added an ethanol solution (10 mL) of MAIm–N (0.28 g, 1.6 mmol) neutralized by 1 M HCl (2.0 mL). The resulting suspension was filtered to obtain colorless precipitates, which was dried under ambient atmosphere to obtain colorless precipitates (0.73 g, yield: 48%). An acetonitrile/ethanol solution (20 mL, 1:1 (*v*/*v*)) of as-prepared **1** (0.03 g) was heated at ca. 343 K for 3 h, and then the supernatant was slowly evaporated at room temperature to obtain colorless plates of **1**. CHN elemental analysis: Calcd for C_30_H_45_N_6_NaMo_8_O_32_: C: 18.53, H: 2.25, N: 4.80%. Found: C: 18.97, H: 2.30, N: 4.98%. IR (KBr disk): 3144 (w), 3109 (w), 3010 (w), 2979 (w), 2927 (w), 2883 (w), 1734 (m), 1718 (m), 1635 (m), 1577 (w), 1543 (w), 1456 (w), 1401 (w), 1362 (w), 1317 (w), 1301 (m), 1170 (m), 1086 (w), 1041 (w), 942 (s), 916 (s), 846 (m), 817 (w), 724 (s), 666 (m), 626 (w), 556 (w), 523 (w) cm^−1^. 

#### 2.2.2. Synthesis of MAImC_8_–Mo_8_

As-prepared MAImC_8_–Mo_8_ (denoted as **2**) was synthesized and recrystallized by a similar procedure to **1**. Na_2_MoO_4_·2H_2_O (0.51 g, 2.1 mmol) dissolved in 10 mL of H_2_O was adjusted to pH 3.8 by 6 M HCl, and then an ethanol solution (10 mL) of MAImC_8_·Br (0.30 g, 0.80 mmol) was added. The resulting suspension was filtered and dried to obtain colorless precipitates (0.38 g, yield: 47%). An acetonitrile/ethanol solution (20 mL, 1:1 (*v*/*v*)) of as-prepared **2** (0.03 g) was heated at ca. 343 K for 3 hours, and the supernatant was slowly evaporated at room temperature to obtain colorless plates of **2**. CHN elemental analysis: Calcd for C_51_H_87_N_6_NaMo_8_O_32_: C: 29.10, H: 4.26, N: 3.99%. Found: C: 28.05, H: 4.14, N: 3.52%. IR (KBr disk): 3136 (w), 3105 (w), 2956 (m), 2925 (s), 2854 (m), 1719 (m), 1636 (w), 1560 (w), 1457 (w), 1404 (w), 1377 (w), 1362 (w), 1317 (w), 1296 (w), 1162 (m), 1039 (w), 943 (s), 913 (s), 844 (m), 714 (s), 666 (m), 555 (w), 525 (w), 473 (w), 453 (w), 442 (w), 416 (w) cm^−1^. 

#### 2.2.3. Synthesis of MAIm–SiW_12_

As-prepared MAIm–SiW_12_ (denoted as **3**) was synthesized by adding a 10 mL ethanol solution containing MAIm–N (0.59 g, 3.3 mmol) to a 10 mL ethanol solution of dodecatungstosilicic acid 26 hydrate (H_4_[SiW_12_O_40_]·26H_2_O (H–SiW_12_), 2.0 g (0.60 mmol)). The obtained suspension was separated by decantation to obtain colorless precipitates, which were washed by water and dried under ambient atmosphere (1.3 g, yield: 51%). A 1,4-dioxane solution (20 mL) of as-prepared **3** (0.03 g) was heated at ca. 333 K for 3 h, and the supernatant was kept at 298 K to obtain colorless plates of **3**. CHN elemental analysis: Calcd for C_36_H_52_N_8_SiW_12_O_48_: C: 12.01, H: 1.46, N: 3.11%. Found: C: 12.03, H: 1.47, N: 3.04%. IR (KBr disk): 3145 (w), 3072 (w), 2963 (w), 2927 (w), 1715 (m), 1634 (w), 1578 (w), 1547 (w), 1449 (w), 1404 (w), 1319 (w), 1297 (w), 1168 (m), 1085 (w), 1013 (w), 973 (s), 922 (s), 884 (w), 796 (s), 665 (w), 622 (w), 534 (w) cm^−1^.

#### 2.2.4. Synthesis of MAImC_8_–SiW_12_

As-prepared MAImC_8_–SiW_12_ (denoted as **4**) was synthesized and recrystallized by a similar procedure to **3**. H–SiW_12_ (1.2 g, 0.36 mmol) was dissolved in 5 mL of ethanol, and then an ethanol solution (10 mL) of MAImC_8_·Br (0.50 g, 1.3 mmol) was added. The resulting suspension was filtered and dried under ambient atmosphere to obtain colorless precipitates (1.2 g, yield: 69%). An 1,4-dioxane/ethanol solution (20 mL, 1:1 (*v*/*v*)) of as-prepared **4** (0.03 g) was heated at ca. 333 K for 3 hours, and the supernatant was kept at 298 K to obtain colorless plates of **4**. CHN elemental analysis: Calcd for C_68_H_116_N_8_SiW_12_O_48_: C: 19.50, H: 2.79, N: 2.74%. Found: C: 20.18, H: 2.89, N: 2.77%. IR (KBr disk): 3142 (w), 3109 (w), 2957 (w), 2925 (m), 2853 (m), 1719 (w), 1635 (w), 1561 (w), 1457 (w), 1401 (w), 1377 (w), 1359 (w), 1318 (w), 1295 (w), 1161 (w), 1107 (w), 1054 (w), 1013 (w), 973 (m), 920 (s), 884 (w), 795 (s), 653 (w), 530 (w), 487 (w), 423 (w) cm^−1^. 

### 2.3. X-ray Crystallography

Single crystal X-ray diffraction measurements for **1**–**3** were performed with an ADSC Q210 CCD area detector by using synchrotron radiation (*λ* = 0.60000–0.80000 Å, Table 1) at 2D beamline in the Pohang Accelerator Laboratory (PAL, a synchrotron radiation facility in Pohang, Republic of Korea). The processing of both diffraction images and absorption correction were performed with HKL3000 [45]. The diffraction measurements for **4** were made on a Rigaku XtaLAB P200 diffractometer (Rigaku Corporation, Tokyo, Japan) using graphite monochromated Mo Kα radiation, and the data were collected and processed using CrysAlisPro [46]. The structures were solved by the dual-space algorithm using SHELXT Version 2014/5 [47] or SHELXS Version 2013/1 [48], and refined by the full-matrix least-squares method on *F*^2^ using SHELXL Version 2014/7 [48]. All calculations were performed using the CrystalStructure software package [49]. Most non-hydrogen atoms were refined anisotropically, and the hydrogen atoms of organic moieties were refined using the riding model. Further details of the crystal structure investigation may be obtained free of charge from the Cambridge Crystallographic Data Centre, 12 Union Road, Cambridge CB2 1EZ, UK; fax: (+44) 1223 336 033; or E-Mail: deposit@ccdc.cam.ac.uk (CCDC 1934917–1934920).

## 3. Results

### 3.1. Mo_8_ hybrids with Polymerizable Ionic Liquids

**Figure 2:**
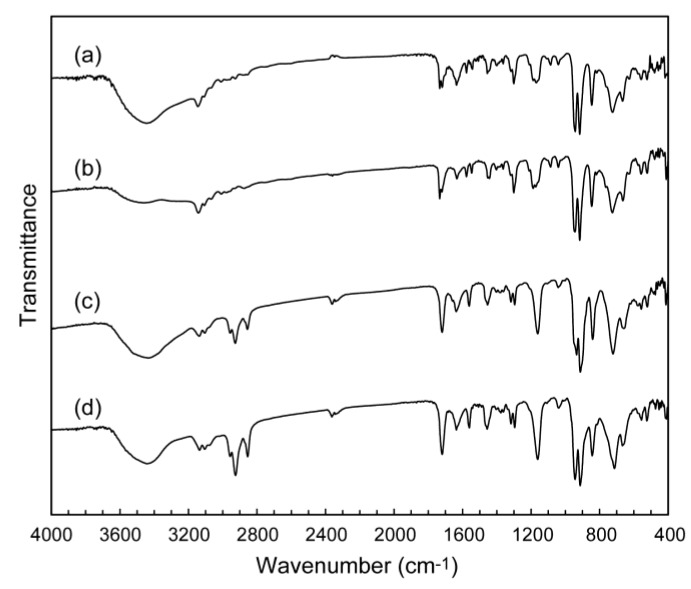
IR spectra of Mo_8_ hybrid crystals: (**a**) as-prepared **1**; (**b**) recrystallized **1**; (**c**) as-prepared **2**; (**d**) recrystallized **2**.

Octamolybdate (Mo_8_) hybrids with the polymerizable ionic liquids of MAIm and MAImC_8_ were obtained as colorless precipitates in ca. 50% yield. IR spectra of both as-prepared MAIm–Mo_8_ (**1**, Figure 2a) and MAImC_8_–Mo_8_ (**2**, Figure 2c) exhibited characteristic peaks of the β-Mo_8_ anion [50,51,52] in the range of 400–1000 cm^−1^ together with the peaks derived from MAIm or MAImC_8_ (methylene groups in 2800–3000 cm^−1^ and methacryloyl group in 1200–1800 cm^−1^), indicating the successful hybridization of Mo_8_ with the polymerizable ionic liquids. 

Both as-prepared precipitates of **1** and **2** were successfully recrystallized by using acetonitrile/ethanol solution to obtain single crystals. The IR spectra of recrystallized **1** (Figure 2b) were almost identical to that of the as-prepared **1** (Figure 2a), indicating that the molecular structures were retained before and after the recrystallization. The molecular structures of **2** were also the same before and after the recrystallization as observed in the IR spectra of hybrid crystal **2** (Figure 2c,d).

**Figure 3:**
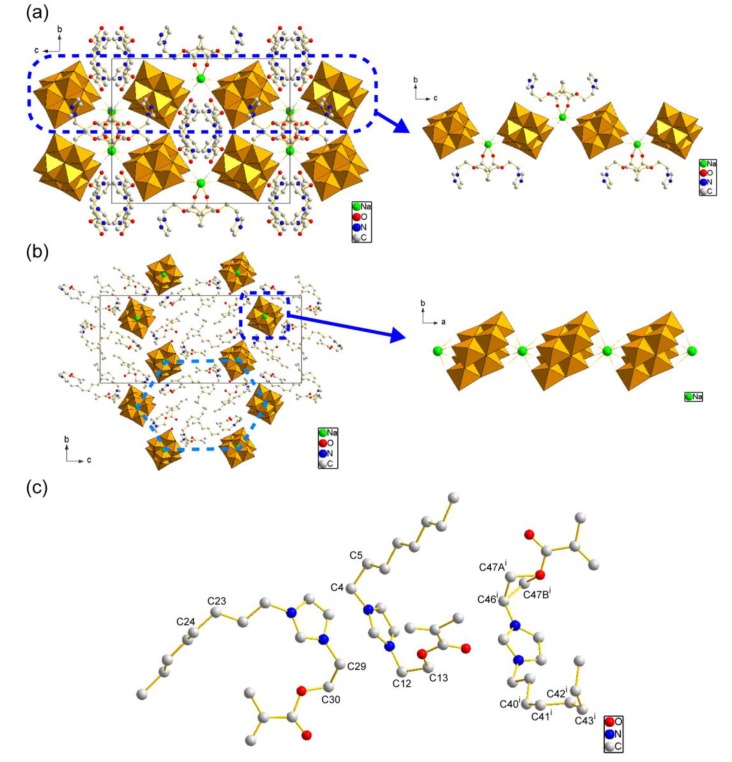
Crystal structures of Mo_8_ hybrid crystals (Na: green, C: gray, N: blue, O: red). Mo_8_ anions are represented by polyhedrons. H atoms are omitted for clarity; (**a**) packing diagram of **1** (left, along *a* axis) and molecular arrangement of the Mo_8_–Na^+^ 1D chain in **1** (right, along *a* axis); (**b**) packing diagram of **2** (left, along *a* axis) and molecular arrangement of Mo_8_-Na^+^ 1D chains in **2** (right, along *c* axis). A honeycomb-like arrangement of Mo_8_–Na^+^ 1D chains is highlighted by a light-blue broken hexagon; (**c**) molecular conformations of crystallographically independent MAImC_8_ cations in **2**. C atoms relevant to the *gauche* conformation are numbered. Symmetry code: (*i*) 1 − *x*, −0.5 + *y*, 0.5 − *z*.

X-ray structure analyses together with CHN elemental analyses revealed that the Mo_8_ hybrid crystals were formulated to be [{CH_2_=C(CH_3_)COO(CH_2_)_2_}C_3_H_4_N_2_]_3_Na[Mo_8_O_26_] for **1** and [{CH_2_=C(CH_3_)COO(CH_2_)_2_}C_3_H_3_N_2_(C_8_H_17_)]_3_Na[Mo_8_O_26_] for **2**, respectively (Table 1, Figure 3). Both hybrid crystals contained three MAIm (1+ charge) or MAImC_8_ (1+ charge) and one Na^+^ associated with one β-type Mo_8_ anion (4− charge), being similar to other hybrid crystals consisting of β-type Mo_8_ anions [42,53,54]. Both crystal structures contained a one-dimensional (1D) infinite chain composed of the Na^+^ cation and Mo_8_ anion (Mo_8_–Na^+^ 1D chain) as shown in Figure 3a,b, while the coordination environments of Na^+^ were different. The Na^+^ cation in **1** was surrounded by O atoms of two Mo_8_ anions and two MAIm cations, and located in a six-fold coordination environment (Na–O distance: 2.28–2.43 Å, mean value: 2.36 Å) to form a zig-zag chain structure (Figure 3a, right). On the other hand, the Na^+^ cation in **2** was sandwiched by only two β-Mo_8_ anions to possess an eight-fold coordination environment (Na–O distance: 2.40−2.90 Å, mean value: 2.60 Å), resulting in a more straight chain structure (Figure 3b, right). 

In addition, the packing manners of the Mo_8_–Na^+^ 1D chains in the crystal structures of **1** and **2** were different. The Mo_8_–Na^+^ 1D chains in **1** were rather densely packed in the crystal structure (Figure 3a, left), while the Mo_8_–Na^+^ 1D chains in **2** were located more separately, to exhibit a honeycomb-like structure along the *a*-axis direction (Figure 3b, left). Namely, **1** had a rather isotropic arrangement of the Mo_8_–Na^+^ 1D chains, while **2** possessed a more anisotropic arrangement of the Mo_8_–Na^+^ 1D chains in the bulk crystal structure. This difference in the arrangements of the Mo_8_–Na^+^ 1D chains between **1** and **2** will be due to the presence of the amphiphilic moiety in the polymerizable ionic liquid cations. The MAImC_8_ had a long alkyl chain, which interacted with itself to control the arrangement of the Mo_8_–Na^+^ 1D chain in the crystal structures of **2** [30,31,32]. As shown in Figure 3c, several C–C bonds in the methylene groups of MAImC_8_ had a *gauche* conformation, which forced both octyl and methacryloyl groups to locate to the same side against the charged and hydrophilic imidazolium ring. These molecular conformations of MAImC_8_ caused the segregation of hydrophilic and hydrophobic parts in the crystal structure of **2**, resulting in the anisotropic honeycomb-like arrangement of the Mo_8_–Na^+^ 1D chains (Figure 3b, left). The octyl chains were not interdigitated in a straight manner.

**Figure 4:**
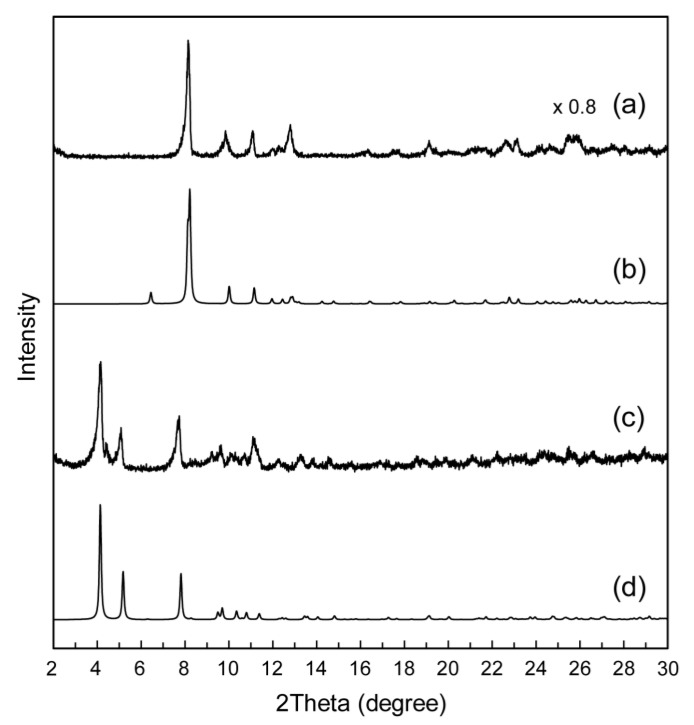
Powder XRD patterns of Mo_8_ hybrid crystals: (**a**) as-prepared **1**; (**b**) calculated pattern of **1** using the structure obtained by single-crystal X-ray diffraction; (**c**) as-prepared **2**; (**d**) calculated pattern of **2** using the structure obtained by single-crystal X-ray diffraction.

Powder XRD patterns of as-prepared **1** and **2** (Figure 4a,c) were quite similar in the peak positions to the patterns calculated from the single crystal structure of **1** and **2** (Figure 4b,d), indicating that the crystal structures of **1** and **2** were retained before and after the recrystallization. Slight differences in the peak intensity and position of the patterns may be derived from the difference in the measurement temperature (powder: ambient temperature, single crystal: 100 K). This suggests that the Mo_8_–Na^+^ 1D chains were already formed in the as-prepared precipitates due to their stable and rigid structures. The amphiphilic moiety of MAImC_8_ in **2** also contributed to the formation of a stable and rigid crystal structure derived from the van der Waals interactions between the octyl chains.

### 3.2. SiW_12_ Hybrids with Polymerizable Ionic Liquids

**Figure 5:**
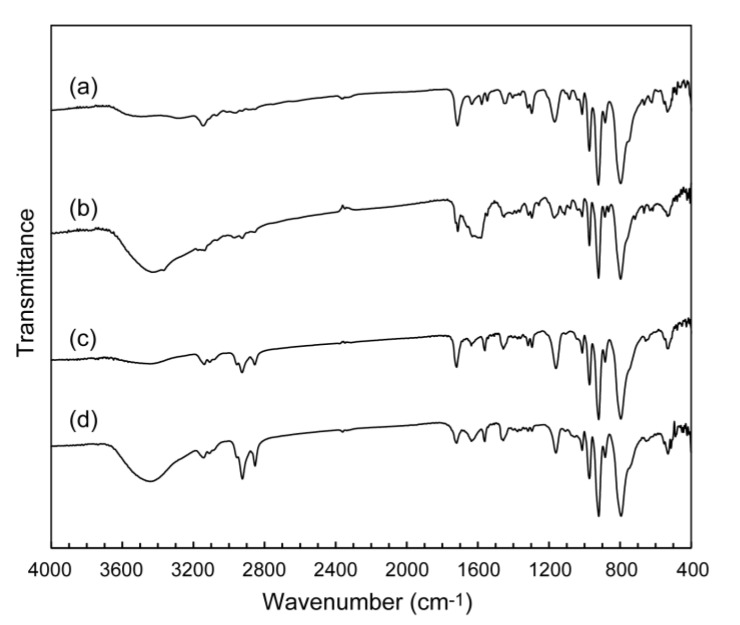
IR spectra of Mo_8_ hybrid crystals: (**a**) as-prepared **3**; (**b**) recrystallized **3**; (**c**) as-prepared **4**; (**d**) recrystallized **4**.

Dodecatungstosilicate (SiW_12_) hybrids with the polymerizable ionic liquids were obtained as colorless precipitates in ca. 50% yield for MAIm–SiW_12_ (**3**) and ca. 70% yield for MAImC_8_–SiW_12_ (**4**), respectively. The characteristic peaks of SiW_12_ [17,44,55] were observed for as-prepared **3** (Figure 5a) and **4** (Figure 5c) in the range of 400–1100 cm^−1^ of IR spectra. The presence of the polymerizable ionic liquids were confirmed by the IR spectra (in the range of 2800–3000 cm^−1^ for methylene groups and 1200–1800 cm^−1^ for methacryloyl group), which showed successful formation of the hybrid crystals comprising SiW_12_ and the polymerizable ionic liquids. 

Single crystals of **3** were obtained from 1,4-dioxane (C_4_H_4_O_2_) solution, while single crystals of **4** were grown from 1,4-dioxane/ethanol solution. As-prepared **3** (Figure 5a) and recrystallized **3** (Figure 5b) were almost the same in the IR spectra, indicating the retention of the molecular structures before and after the recrystallization. A slight difference in the peaks in the range of 1500–1700 cm^−1^ may be derived from the difference in the crystal structures of as-prepared **3** and recrystallized **3** (see below). The retention of the molecular structures of **4** was also verified by the IR spectra measured before and after the recrystallization (Figure 5c,d).

**Figure 6:**
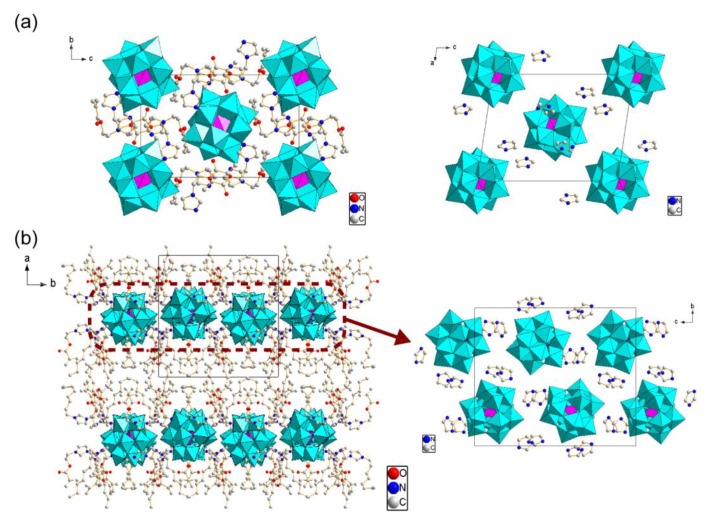
Crystal structures of SiW_12_ hybrid crystals (C: gray, N: blue, O: red). SiW_12_ anions are represented by polyhedrons. H atoms are omitted for clarity; (**a**) packing diagram of **3** (left, along *a* axis) and molecular arrangement of SiW_12_ anions in **3** (right, along *b* axis); (**b**) packing diagram of **4** (left, along *c* axis) and molecular arrangement of SiW_12_ anions in **4** (right, along *a* axis).

Chemical formulae were revealed to be [{CH_2_=C(CH_3_)COO(CH_2_)_2_}C_3_H_4_N_2_]_4_[SiW_12_O_40_]·C_4_H_4_O_2_ for **3** and [{CH_2_=C(CH_3_)COO(CH_2_)_2_}C_3_H_3_N_2_(C_8_H_17_)]_4_[SiW_12_O_40_]·C_4_H_4_O_2_·H_2_O for **4**, respectively (Table 1). These hybrid crystals contained four polymerizable ionic liquid cations (1+ charge) associated with one SiW_12_ anion (4− charge). All of the protons of the starting H–SiW_12_ were replaced by ion-exchange reactions without remaining counter cations after the hybridization [44,56,57,58], which was different from the cases of Mo_8_ hybrid crystals of **1** and **2**. Both recrystallized SiW_12_ hybrid crystals of **3** and **4** contained the solvents of crystallization (1,4-dioxane utilized in the recrystallization process for **3**; 1,4-dioxane and water for **4**), which also contrasts with the Mo_8_ hybrid crystals of **1** and **2**. Figure 6 depicts crystal structures of **3** (Figure 6a) and **4** (Figure 6b). In both crystals, each SiW_12_ anion was isolated by the imidazolium moieties of the polymerizable ionic liquids (Figure 6a, right for **3**; Figure 6b, right for **4**). 

The molecular arrangements of the SiW_12_ anions in the bulk crystal structures of **3** and **4** were quite different. The SiW_12_ anions are arranged rather densely and isotropically in **3** (Figure 6a, left). On the contrary, the SiW_12_ anions in **4** formed the inorganic monolayers sandwiched by the MAImC_8_ organic layers with an interlayer distance of 20.2 Å (Figure 6b, left), and exhibited much more anisotropic structure than **3**. Such anisotropic molecular arrangement will be induced by the amphiphilic moiety of the MAImC_8_ cations. The MAImC_8_ in **4** had similar conformations to those observed in **2** (Figure 3c). The octyl chains in **4** were not interdigitated in a straight manner as in the crystals of **2**. 

**Figure 7:**
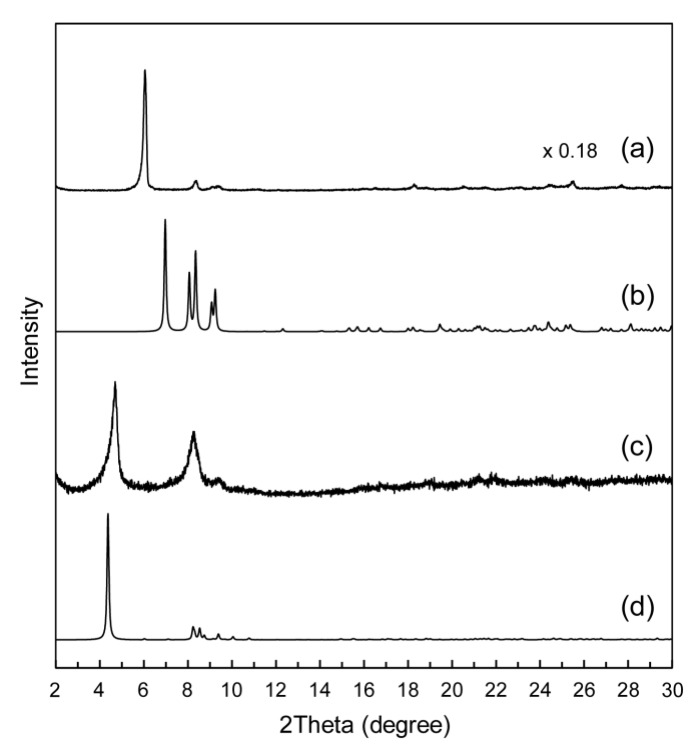
Powder XRD patterns of SiW_12_ hybrid crystals: (**a**) as-prepared **3**; (**b**) calculated pattern of **3** using the structure obtained by single-crystal X-ray diffraction; (**c**) as-prepared **4**; (**d**) calculated pattern of **4** using the structure obtained by single-crystal X-ray diffraction.

The powder XRD pattern of as-prepared **3** (Figure 7a) was different from that calculated from the single crystal structure of **3** (Figure 7b). This demonstrates that the crystal structures of **3** changed after the recrystallization, probably because of desolvation of solvent molecules of the crystals under the ambient atmosphere. On the other hand, powder XRD patterns of as-prepared **4** (Figure 7c) were essentially similar to the patterns calculated from the single crystal structure of **4** (Figure 7d), indicating that the crystal structures of **4** were retained before and after the recrystallization. These results suggest that the amphiphilic moiety of the polymerizable ionic liquid enabled formation of the stable crystal structures of **4** owing to the van der Waals interactions between the octyl chains.

## 4. Discussion

As observed in the crystal structures of **2** and **4**, introducing the amphiphilic moiety into the polymerizable ionic liquid enabled the construction of the anisotropic molecular arrangements of POM inorganic clusters in the bulk crystal structures. The amphiphilic MAImC_8_ cation behaved as a structure-directing reagent, like typical surfactants [30,31,32], owing to the van der Waals interactions between the octyl chains of MAImC_8_. The presence of a long alkyl chain in the polymerized ionic liquids induced the segregation of hydrophilic parts (charged POM and imidazolium ring) and a hydrophobic moiety (alkyl chain and methacryloyl group), which led to the formation of the anisotropic POM arrangements in the crystal structures of **2** and **4**. Introducing the amphiphilic moiety into the polymerizable ionic liquid also realized stable crystal structures. The crystal structures of **2** and **4** were retained before and after the recrystallization process, although the crystal structures of the hybrid crystal of **3** without the amphiphilic moiety changed in their crystal structures through the recrystallizations procedures. 

The β-type Mo_8_ anion tends to coordinate metal cations to form a 1D chain or two-dimensional (2D) layered structures [42,54]. The smaller metal cations such as Na^+^ or Ag^+^ preferred the 1D chain structure, while the larger cations such as K^+^ or Cs^+^ gave rise to a 2D layered structure. In the case of **1** and **2**, the Na^+^ cation of the starting material (Na_2_MoO_4_·2H_2_O) remained to form the Mo_8_–Na^+^ 1D chain structures in the crystal structures. Furthermore, **1** and **2** kept their molecular and crystal structures before and after the recrystallization (Figure 2 and Figure 4), suggesting the retention of the Mo_8_–Na^+^ 1D chain structures. These stable Mo_8_–Na^+^ 1D chain structures could be beneficial to the Na^+^-conducting materials. On the other hand, the typical Keggin-type SiW_12_ anion exhibits *T_d_* symmetry, which is higher than the *C*_2*h*_ symmetry of the β-Mo_8_ anion (Figure 1b) [59,60]. The more spherical SiW_12_ anion tends to be surrounded by the heterocyclic moiety of the polymerizable ionic liquid or surfactant [44,56], resulting in the isolated arrangement of SiW_12_ without the coordination by metal cations.

As described here, introducing the amphiphilic moiety into the polymerizable ionic liquid is an effective way to control the POM arrangements in the hybrid single crystals. Such controlled arrangement of POM anions will have beneficial emergent functions such as conductive properties [12,13,29]. In principle, these POM hybrid crystals of **1**–**4** behave as inorganic–organic hybrid monomers to construct hybrid polymers [20,42,43,44], which could pave the way to another category of conductive materials. The polymerization of these hybrids and investigation of conductivities are in progress. 

## 5. Patents

A Japanese patent (JP 2018012758 A) resulted from the work reported in this manuscript (T.I., Y.N., S.K., M.H., Y.O.).

## Figures and Tables

**Table 1:** Crystallographic data of the hybrid crystals.

Compound	1	2	3	4
Chemical formula	C_27_H_39_N_6_NaMo_8_O_32_	C_51_H_87_N_6_NaMo_8_O_33_	C_40_H_60_N_8_SiW_12_O_50_	C_72_H_107_N_8_SiW_12_O_51_
Formula weight	1750.14	2102.78	3687.22	4134.95
Crystal system	monoclinic	orthorhombic	monoclinic	monoclinic
Space group	*I*2/*a* (No. 15)	*P*2_1_2_1_2_1_ (No. 19)	*P*2_1_/*n* (No. 14)	*P*2_1_/*c* (No. 14)
*a* (Å)	13.6080(3)	9.33330(10)	14.8460(3)	20.7451(16)
*b* (Å)	17.6620(4)	18.6205(2)	12.6020(3)	21.2737(6)
*c* (Å)	21.8453(5)	42.7175(5)	19.6940(5)	25.5199(8)
*α* (°)	90.0000	90.0000	90.0000	90.0000
*β* (°)	95.0422(14)	90.0000	98.6520(10)	103.067(5)
*γ* (°)	90.0000	90.0000	90.0000	90.0000
*V* (Å^3^)	5230.1(2)	7423.90(14)	3642.61(15)	10970.9(10)
*Z*	4	4	2	4
*ρ*_calcd_ (g cm^−3^)	2.222	1.881	3.361	2.503
*T* (K)	100	100	100	100
Wavelength (Å)	0.80000	0.63000	0.60000	0.71075
*μ* (mm^−1^)	2.725	0.976	12.044	12.638
No. of reflections measured	35127	102284	94446	106869
No. of independent reflections	5143	15761	14513	24752
*R*_int_	0.0600	0.0800	0.0870	0.1418
No. of parameters	394	883	507	644
*R*_1_ (*I* > 2*σ*(*I*))	0.0553	0.0564	0.0721	0.0777
*wR*_2_ (all data)	0.1680	0.1576	0.2046	0.2256

## Supplementary Materials

The following are available online at https://www.mdpi.com/1996-1944/12/14/2283/s1, synthetic procedures of polymerizable ionic liquids, Figure S1: Synthetic route of MAIm, cif files of **1**–**4**.
