# Supplementary material for: Dimensional Control in Polyoxometalate Crystals Hybridized with Amphiphilic Polymerizable Ionic Liquids
Source: Materials (Basel). 2019 Jul 16;12(14):2283. doi: 10.3390/ma12142283 (PMC6678579; doi:10.3390/ma12142283)
Supplement: Supplementary file 1 [file materials-12-02283-s001.pdf]

materials

Supporting Information

Dimensional Control in Polyoxometalate Crystals Hybridized with Amphiphilic Polymerizable Ionic Liquids

Toshiyuki Misawa [1], Jun Kobayashi [1], Yoshiki Kiyota [1], Masayuki Watanabe [2], Seiji Ono [2], Yosuke Okamura [2], Shinichi Koguchi [1], Masashi Higuchi [2], Yu Nagase [2] and Takeru Ito [1,*]

[1] Department of Chemistry, School of Science, Tokai University, 4-1-1 Kitakaname, Hiratsuka 259-1292, Japan
[2] Department of Applied Chemistry, School of Engineering, Tokai University, 4-1-1 Kitakaname, Hiratsuka 259-1292, Japan
* Correspondence: takeito@keyaki.cc.u-tokai.ac.jp; Tel.: +81-463-58-1211 ex. 3737 (T.I.)

Received: 19 June 2019; Accepted: 15 July 2019; Published: date

1. Syntheses of polymerizable ionic-liquids

1.2. Synthesis of 2-(1H-Imidazol-1-yl) Ethyl Methacrylate (MAIm)

1.2.1. Synthesis of 1-(2-Chloroethyl)-1H-Imidazole

The solution of imidazole 2.04 g (30 mmol) in 1,2-dichloroethane (30 mL) was added to tetrabutylammonium bromide 0.20 g (0.6 mmol) and K_2CO_3 0.82 g (6 mmol). The result mixture was stirred at 358 K for 5 h. The mixture was extracted with $CHCl_3$, and the organic layer was washed with water, dried ($MgSO_4$) and evaporated. The product was isolated by silica gel column chromatography to give the title compound (1.47 g, 37%) as a colorless liquid.

^{1}H NMR: δ (500 MHz, $CDCl_3$, ppm): 3.76 (2H, t, *J* = 10 Hz), 4.28 (2H, t, *J* = 10 Hz), 6.98 (1H, s), 7.10 (1H, s), 7.54 (1H, s).

1.2.2. Synthesis of 2-(1H-Imidazol-1-yl) Ethyl Methacrylate (MAIm)

The solution of 1-(2-chloroethyl)-1*H*-imidazole 1.13 g (13.2 mmol) in THF (25 mL) was added to methacrylic acid 1.47 g (11.2 mmol) and K_2CO_3 3.58 g (25.9 mmol). The resulting mixture was stirred at 339 K overnight. The mixture was extracted with $CHCl_3$, and the organic layer was washed with water, dried ($MgSO_4$), and evaporated. The product was isolated by silica gel column chromatography to give 1.70 g (84%) of the title compound as a colorless liquid.

^{1}H NMR: δ (500 MHz, $CDCl_3$, ppm): 1.93 (3H, s), 4.25 (2H, t, *J* = 5 Hz), 4.40 (2H, t, *J* = 5 Hz), 5.61 (1H, s), 6.10 (1H, s), 6.96 (1H, s), 7.08 (1H, s), 7.51 (1H, s).

1.2. Synthesis of 1-(2-(methacryloyloxy) ethyl)-3-octyl-1H-imidazol-3-ium Bromide (MAImC$_8$-Br)

The solution of 2-(1*H*-imidazol-1-yl) ethyl methacrylate (MAIm, 0.61 g (3.4 mmol)) in 1-bromooctane 1.15 g (6 mmol) was stirred at 333 K overnight. The product was isolated by silica gel column chromatography to give 1.00 g (80%) of the title compound as a yellow liquid.

^{1}H NMR: δ (500 MHz, DMSO-d_6), ppm): 0.86 (3H, t, *J* = 6.91 Hz), 1.25 (10H, m), 1.77 (2H, m), 1.85 (3H, t, *J* = 1.14 Hz), 4.19 (2H, t, *J* = 6.93 Hz), 4.48 (2H, m), 4.54 (2H, t, *J* = 4.41 Hz,), 5.72 (1H, quint., *J* = 1.61 Hz), 6.03 (1H, t, *J* = 1.13 Hz), 7.85 (2H, m), 9.29 (1H, s, 1H).

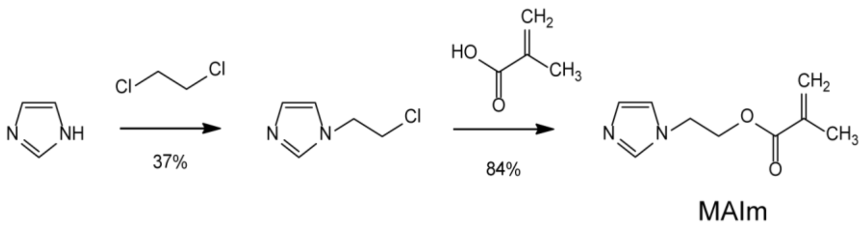

Figure S1. Synthetic route of MAIm.

© 2019 by the authors. Submitted for possible open access publication under the terms and conditions of the Creative Commons Attribution (CC BY) license (http://creativecommons.org/licenses/by/4.0/).
